# A Gut Feeling: An Exploratory Multi-Omics Study of Gut Microbiome Dysbiosis and Metabolome and Lipidome Alterations in GATA2 Deficiency

**DOI:** 10.3390/ijms27104294

**Published:** 2026-05-12

**Authors:** Samuele Roncareggi, Francesca Fioredda, Katia Girardi, Simone Serrao, Giulia Capitoli, Rebecca Fumagalli, Marta Nobile, Grazia Fazio, Fabiola Guerra, Maria Grazia Valsecchi, Stefano Rebellato, Marika Casillo, Maria Rosaria Fantuz, Giovanni Savarese, Giuseppe Paglia, Eleonora Gambineri, Adriana Cristina Balduzzi, Andrea Biondi, Francesco Saettini

**Affiliations:** 1 Dipartimento di Medicina e Chirurgia, Università degli Studi Milano-Bicocca, 20900 Monza, Italy; s.roncareggi@campus.unimib.it (S.R.); simone.serrao@unimib.it (S.S.); m.nobile13@campus.unimib.it (M.N.); giuseppe.paglia@unimib.it (G.P.);; 2 Unit of Hematology, Istituto di Ricovero e Cura a Carattere Scientifico (IRCCS) G. Gaslini, 16147 Genoa, Italy; francescafioredda@gaslini.org; 3 Department of Pediatric Hematology/Oncology, Cell and Gene Therapy, Istituto di Ricovero e Cura a Carattere Scientifico (IRCCS) Bambino Gesù Children’s Hospital, 00165 Rome, Italy; 4 Bicocca Bioinformatics Biostatistics and Bioimaging (B4) Centre, School of Medicine and Surgery, University of Milano-Bicocca, 20126 Monza, Italy; giulia.capitoli@unimib.it (G.C.); grazia.valsecchi@unimib.it (M.G.V.); 5 Biostatistics and Clinical Epidemiology, Fondazione Istituto di Ricovero e Cura a Carattere Scientifico (IRCCS) San Gerardo dei Tintori, 20900 Monza, Italy; 6 Centro Tettamanti, Fondazione Istituto di Ricovero e Cura a Carattere Scientifico (IRCCS) San Gerardo dei Tintori, 20900 Monza, Italy; 7 Pediatria, Fondazione Istituto di Ricovero e Cura a Carattere Scientifico (IRCCS) San Gerardo dei Tintori, 20900 Monza, Italy; 8 Ames Centro Polidiagnostico Strumentale, 80013 Casalnuovo di Napoli, Italy; 9 Department of Pediatric Oncology/Hematology, Meyer Children’s Hospital Istituto di Ricovero e Cura a Carattere Scientifico (IRCCS), 50139 Florence, Italy; 10 Department of Neurosciences, Psychology, Drug Research and Child Health (NEUROFARBA), University of Florence, 50139 Florence, Italy

**Keywords:** GATA2, microbiome, immunodeficiency, myelodysplastic syndrome, inborn errors of immunity

## Abstract

GATA2 deficiency predisposes patients to recurrent infections, myelodysplastic neoplasms (MDSs), and malignancies through disrupted hematopoiesis and immune dysfunction. The role of the gut microbiome (GM) in this condition remains poorly defined. In this multicenter study, we analyzed GM composition, metabolomic, and lipidomic profiles in 12 Italian GATA2-deficient patients, comparing non-HSCT and post-HSCT GATA2-deficient individuals with healthy controls. Non-HSCT patients showed a relative enrichment of Proteobacteria-associated Gram-negative taxa, accompanied by increased levels of metabolites and lipids previously associated with inflammatory processes. Post-HSCT patients displayed profiles with a trend toward partial normalization of GM composition and metabolic features. Overall, our findings suggest the presence of microbiome and metabolic patterns in GATA2-deficient patients, which may reflect underlying immune and hematopoietic alterations, although these observations should be interpreted as descriptive and require validation in larger cohorts.

## 1. Introduction

GATA2 is a transcription factor critical for hematopoietic stem cell maintenance and differentiation. GATA2 deficiency lies at the crossroads of inborn errors of immunity (IEI) and myelodysplastic syndromes (MDSs), profoundly impacting hematopoiesis and immunity.

Clinically, GATA2 deficiency is a highly heterogeneous disorder characterized by variable age at onset, incomplete penetrance, and a broad spectrum of disease manifestations [1]. Clinical presentation ranges from asymptomatic individuals to severe, life-threatening conditions [2,3,4,5]. The most common features include recurrent infections and hematologic abnormalities. Severe infections occur in up to 80% of patients, with viral infections reported in approximately 70%, particularly human papillomavirus (HPV)-related disease (~60%) and herpesvirus infections (~35%), as well as increased susceptibility to nontuberculous mycobacteria and fungal pathogens. Hematologic manifestations are prominent and include cytopenias, bone marrow hypocellularity, and myeloid neoplasms, observed in 74–81% of patients [2,3,4,5]. Notably, GATA2 deficiency represents the most common germline predisposition to pediatric myelodysplastic syndrome (MDS) [6]. Additional clinical features include lymphedema (11–15%), pulmonary alveolar proteinosis (3.8–18%), sensorineural hearing loss (1.3–43%), and a wide range of dermatologic, neurologic, vascular, and genitourinary abnormalities [2,3,4,5].

The human gut microbiome (GM) has been associated with immune homeostasis and hematopoietic regulation [7,8]; however, its role in IEI remains poorly defined and difficult to disentangle from disease-related and environmental factors. Given the complexity of host–microbiome interactions, particularly in rare genetic disorders, microbiome analyses in this context are best interpreted as descriptive and hypothesis-generating.

In this multicentric study, we aimed to explore whether reproducible microbiome-associated patterns could be identified in 12 Italian GATA2-deficient patients.

## 2. Results

Twelve patients (age range 19−46 years, M:F = 5:7) with genetically confirmed GATA2 deficiency [9,10,11] were enrolled and compared with 24 age-matched HD (age range 14−44, M:F = 9:15). Inclusion criteria required the absence of antibiotic treatment in the preceding three months. Patients were stratified according to hematopoietic stem cell transplantation (HSCT) status into non-HSCT and post-HSCT groups. Five patients had not undergone HSCT (non-HSCT group), while seven were evaluated after HSCT (post-HSCT group; median time from HSCT: 8 years, range 3–16 years). At the time of sampling, no patients had AML or were receiving active chemotherapy. Non-HSCT patients were included across different disease stages (e.g., asymptomatic, prior AML in remission or MDS), reflecting the clinical heterogeneity of GATA2 deficiency. Additional criteria for the post-HSCT group included the absence of chronic graft-versus-host disease (GVHD), full donor chimerism, and no immunosuppressive treatment for at least six months prior to enrollment. Clinical characteristics of the included patients are summarized in Table 1. No significant differences were observed in age or gender distribution between groups (HD, non-HSCT and post-HSCT groups; Table S1).

**Figure 1:**
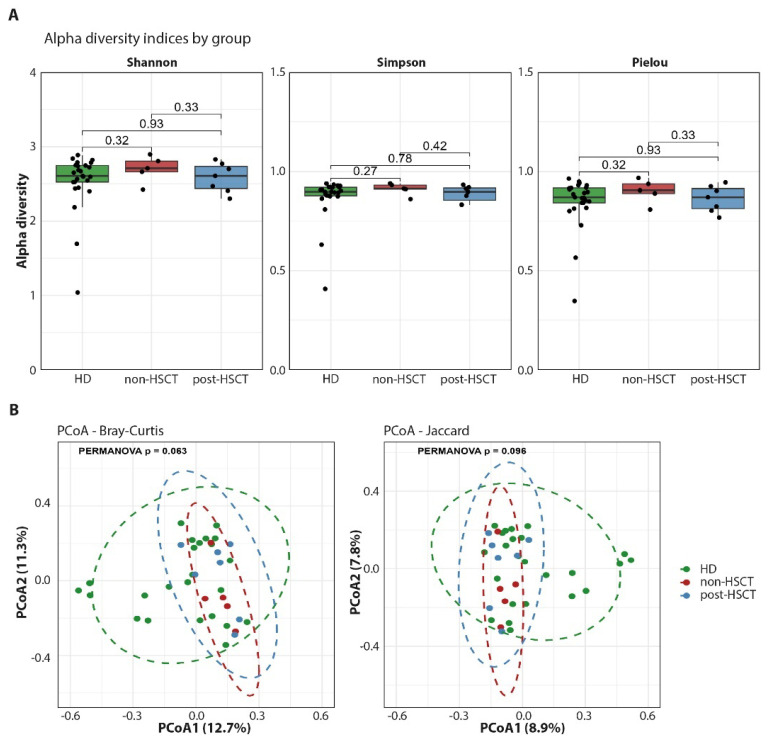
Association of GATA2 deficiency with microbiome signature. Alpha diversity analyses based on Shannon, Simpson and Pielou indices (**A**). Principal coordinates analysis (PCoA) plots of beta diversity based on the Bray–Curtis and Jaccard metrics (**B**).

We examined whether GATA2 deficiency, either before or after HSCT, was associated with changes in GM alpha and beta diversity (Figure 1). Alpha diversity refers to metrics that quantify species richness, evenness, and overall diversity within a single sample. In contrast, beta diversity assesses differences in community composition by comparing the similarity or dissimilarity between two or more samples [12]. Alpha diversity was assessed using Shannon, Simpson and Pielou’s indices, while beta diversity was assessed using Bray–Curtis and Jaccard metrics. Comparisons between HD and non-HSCT or post-HSCT groups using Shannon, Simpson, and Pielou indices revealed no significant differences in overall diversity, dominance, or community evenness. Beta-diversity analysis based on Bray–Curtis and Jaccard distances demonstrated a trend toward group separation that did not reach statistical significance (PERMANOVA: Bray–Curtis *p* = 0.063; Jaccard *p* = 0.098). The stronger trend observed with Bray–Curtis suggests that potential group differences may be driven by changes in the relative abundance of shared taxa rather than by presence–absence patterns.

Despite the absence of significant differences in global diversity metrics, compositional differences in GM were observed at multiple taxonomic levels.

**Figure 2:**
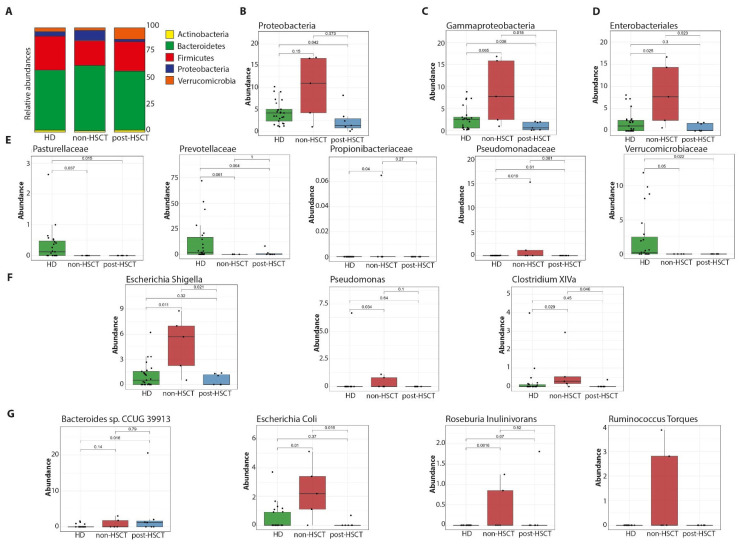
Gut microbiome signatures distinguish patients with GATA2 deficiency from healthy individuals. Relative abundance of phyla (**A**). Differentially abundant phyla (**B**), classes (**C**), orders (**D**), families (**E**), genera (**F**) and species (**G**).

Non-HSCT GATA2-deficient patients showed a trend toward increased relative abundance of Proteobacteria compared with HD and post-HSCT patients (*p* = 0.15 and *p* = 0.73, respectively; Figure 2A,B). Within the Proteobacteria phylum, a similar trend was observed for Gammaproteobacteria (*p* = 0.065 and *p* = 0.018; Figure 2C). Non-HSCT patients displayed significantly higher abundance of Enterobacterales compared with both HD and post-HSCT groups (*p* = 0.01 and *p* = 0.013, respectively; Figure 2D). At the family level, Pseudomonadaceae were significantly increased in non-HSCT patients compared with HD (*p* = 0.019; Figure 2E). In contrast, HD exhibited a higher relative abundance of Pasteurellaceae and Verrucomicrobiaceae compared with GATA2-deficient patients, including both non-HSCT and post-HSCT groups (Pasteurellaceae: *p* = 0.037 and 0.15; Verrucomicrobiaceae: *p* = 0.05 and 0.022, respectively). HD also showed a trend toward increased Prevotellaceae compared with non-HSCT patients (*p* = 0.061). At the genus level, non-HSCT patients were characterized by an overrepresentation of *Escherichia*/*Shigella*, *Pseudomonas*, and *Clostridium XIVa* (Figure 2F). Species-level analysis further revealed a significant enrichment of *Bacteroides* sp. CCUG 39913, *Escherichia coli*, *Roseburia inulinivorans*, and *Ruminococcus torques* in non-HSCT individuals compared with HD (Figure 2G).

**Figure 3:**
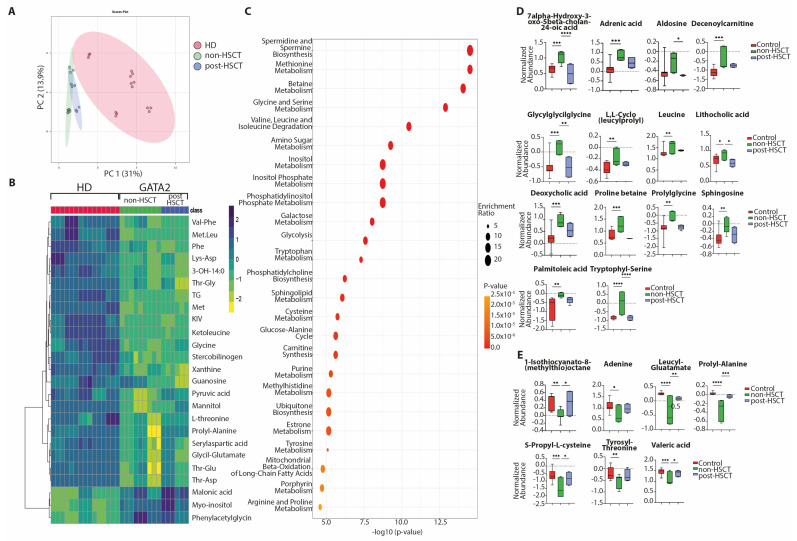
Metabolomic changes in GATA2 deficiency. Principal coordinates analysis (PCoA) (**A**) comparing patients with GATA2 deficiency (n = 5) and healthy individuals (HD; n = 5). Heatmap of top 25 metabolites considered based on *t*-test (**B**) of the metabolomic profiles distinguishing healthy individuals (HD; n = 5) from patients with GATA2 deficiency. Enrichment analysis of top 25 enriched metabolic pathways in non-HSCT compared to HD (**C**). Box plots showing metabolites increased (**D**) and decreased (**E**) in non-HSCT individuals. In the heatmap, rows display metabolites, and columns represent samples (yellow = decreased, dark blue = increased). The brightness of each color corresponds to the magnitude of the difference when compared with average values. *p*-values are indicated wherever required, and *p* < 0.05 is considered significant. * *p* < 0.05; ** *p* < 0.01; *** *p* < 0.001; **** *p* < 0.0001. Each sample was analyzed in three technical replicates, as shown in panels (**A**,**C**). 3-OH-14:0 = 3-hydroxytetradecanoic acid. KIV = alpha-ketoisovaleric acid. Lys-Asp = lysilaspartic acid. Met = methionine. Met-Leu = methionil-leucine. Phe = phenylalanine. TG = trigonelline. Thr-Asp = threonyl-aspartic acid. Thr-Gly = threonylglycine. Thr-Glu = threonyl-glutamic acid Val-Phe = valylphenylalanine.

**Figure 4:**
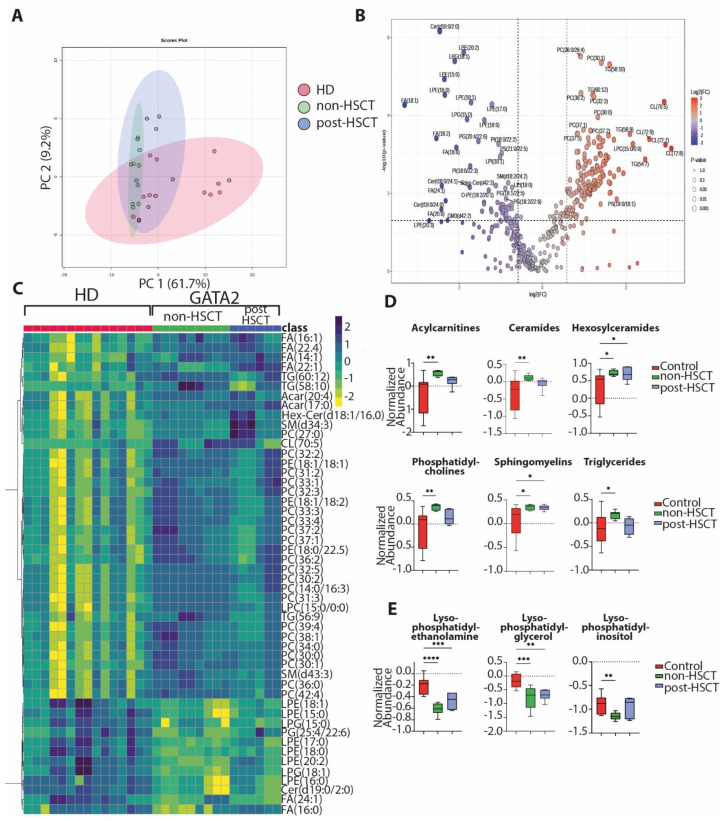
Lipidomic changes in GATA2 deficiency. Principal coordinates analysis (PCoA) plot (**A**) of the lipidomic profiles distinguishing healthy individuals from patients with GATA2 deficiency. Volcano plot (**B**) comparing non-HSCT GATA2 patients with healthy individuals. Heatmap of differentially abundant lipids considered based on *t*-test (**C**) of the lipidomic profiles distinguishing healthy individuals from patients with GATA2 deficiency. Box plots showing metabolites increased (**D**) and decreased (**E**) in non-HSCT individuals. The lines in the volcano plots indicate the significance cutoff for *p*-value (*p* < 0.05) and fold change (FC) (log2FC > 1.5, log2FC < −1.5). All significantly different metabolites are shown. In the heatmap, rows display metabolites, and columns represent samples (yellow = decreased, dark blue = increased). The brightness of each color corresponds to the magnitude of the difference when compared with average values. *p*-values are indicated wherever required, and *p* < 0.05 is considered significant. * *p* < 0.05; ** *p* < 0.01; *** *p* < 0.001; **** *p* < 0.0001. Each sample was analyzed in three technical replicates, as shown in panels (**A**,**C**).

To begin exploring the functional implications of these microbiomes, fecal metabolomic (Figure 3) and lipidomic (Figure 4) profiles were analyzed in non-HSCT (n = 3) and post-HSCT (n = 2) GATA2-deficient patients and compared with HD (n = 5).

Untargeted metabolomic analysis identified a total of 253 polar metabolites. Principal component analysis (PCoA) of fecal samples from GATA2-deficient patients suggested distinct metabolic profiles compared with HD (Figure 3A). Notably, post-HSCT patients exhibited fecal metabolomes that were more homogeneous and less diverse than those of non-HSCT patients, showing partial overlap with HD samples (Figure 3B,C and Figure S1). This pattern mirrored the normalization trends observed in GM composition following HSCT. At the metabolite level, fecal samples from non-HSCT GATA2 patients showed significantly increased levels of several compounds, including 7α-hydroxy-3-oxo-5β-cholan-24-oic acid, adrenic acid, aldosine, decenoylcarnitine, deoxycholic acid, glycylglycylglycine, cyclo(L-leucyl–L-proline), leucine, lithocholic acid, palmitoleic acid, proline betaine, prolylglycine, sphingosine, and tryptophylserine. Conversely, non-HSCT patients exhibited reduced levels of 1-isothiocyanato-8-(methylthio)octane, adenine, leucylglutamate, prolyl-alanine, S-propyl-L-cysteine, tyrosyl-threonine, and valeric acid compared with HD and post-HSCT individuals (Figure 3D,E). Collectively, pathway enrichment analysis comparing non-HSCT GATA2-deficient patients to HD suggested broad metabolic reprogramming involving amino acid metabolism (including methionine, glycine–serine, branched-chain amino acids, cysteine, arginine–proline, tyrosine, and tryptophan pathways), carbohydrate and energy metabolism (glycolysis, amino sugar, galactose, inositol and inositol phosphate pathways), lipid and membrane-associated processes (phosphatidylcholine and sphingolipid metabolism, phosphatidylinositol phosphate metabolism, carnitine synthesis, and mitochondrial β-oxidation of long-chain fatty acids), as well as nucleotide- and heme-related pathways (purine, methylhistidine, and porphyrin metabolism; Figure 3C).

Untargeted lipidome analysis identified 442 lipids. Overall, fecal samples from GATA2-deficient patients showed lipidomic profiles largely comparable to those of HD (Figure 4A). However, several lipids were differentially represented in non-HSCT patients. Consistent with the metabolomic findings, a partial normalization of lipid profiles was observed in post-HSCT patients (Figure 4B–E). Fecal samples from non-HSCT GATA2-deficient patients exhibited significantly increased levels of acylcarnitines (ACar), hexosylceramides (Hex-Cer), phosphatidylcholines (PCs), sphingomyelins (SMs), and triglycerides (TGs). In contrast, non-HSCT patients showed significantly reduced levels of lysophosphatidylethanolamine (LPE), lysophosphatidylglycerol (LPG), and lysophosphatidylinositol (LPI) compared with HD and post-HSCT patients.

## 3. Discussion

To the best of our knowledge, this study provides the first exploratory characterization of GM composition and its potential functional implications in GATA2 deficiency.

In this study, similarity between groups was assessed using beta-diversity metrics (Bray–Curtis and Jaccard distances), while group homogeneity was inferred from the dispersion and clustering of samples in ordination analyses. Compositional skewing was defined as relative enrichment or depletion of specific taxa across taxonomic levels. However, the limited sample size precludes a robust definition of group-level microbiome configurations. Given the complexity of host–microbiome interactions, our findings should be interpreted as descriptive and hypothesis-generating rather than providing mechanistic insight into GATA2 pathogenesis. No significant differences in GM alpha diversity were observed between GATA2-deficient patients and HD, either before or after HSCT. Beta-diversity analyses showed a trend toward group separation but did not reach statistical significance. Alpha diversity reflects within-sample microbial richness and evenness, providing an overall estimate of ecosystem complexity, whereas beta diversity captures differences in community composition between groups, highlighting shifts in relative abundance or the presence of specific taxa. This lack of significant diversity changes in GATA2 deficiency contrasts with findings in other immunodeficiencies [13,14,15,16] and hematologic disorders, such as MDS [17,18] or myeloproliferative neoplasms [19], where reduced microbial diversity and altered microbiota composition are commonly observed. In GATA2 deficiency, our data suggest that global microbiome diversity may be relatively preserved, underscoring potential differences in how this condition impacts the gut ecosystem.

Although overall diversity remained unchanged, distinct compositional shifts were observed in non-HSCT GATA2-deficient patients, suggesting compositional alterations consistent with a potentially pro-inflammatory GM profile. The GM of non-HSCT GATA2-deficient patients showed an overrepresentation of pro-inflammatory taxa, with consistent enrichment across taxonomic levels from the phylum (Proteobacteria) to the species level. Proteobacteria, one of the most abundant phyla in the human GM, are gram-negative bacteria with the outer membrane mainly composed of lipopolysaccharides, a potent immune stimulator. An expansion of Proteobacteria has been proposed as a possible microbial signature of dysbiosis, being associated with inflammatory conditions [20]. Consistent with this, we observed downstream taxonomic expansions along the phylogenetic hierarchy in non-HSCT patients. For instance, the class Gammaproteobacteria (which includes many pathogenic Gram-negatives) and the order Enterobacterales were significantly enriched in non-HSCT patients compared to both HD and post-HSCT groups. At finer taxonomic levels, non-HSCT GATA2 patients showed an overabundance of specific pro-inflammatory bacteria. The Enterobacteriaceae family showed a marked increase in *Escherichia*/*Shigella* in non-HSCT individuals. The genus *Pseudomonas* (family Pseudomonadaceae) was also significantly elevated in non-HSCT patients compared to HD. Additionally, we noted higher representation of *Clostridium XIVa* and other anaerobic taxa commonly associated with dysbiosis. Species-level analysis revealed that non-transplanted patients were specifically enriched in organisms, such as *Escherichia coli*, *Bacteroides* sp. (CCUG 39913), *Roseburia inulinivorans*, and *Ruminococcus torques*, relative to controls. Many of these taxa have pro-inflammatory or opportunistic pathogenic potential [21,22], further supporting a potentially dysbiotic gut environment in non-transplanted GATA2 patients. These findings may represent a descriptive microbiome signature associated with disease or treatment status, rather than a direct contributor to disease mechanisms.

These findings partially resemble observations in other contexts of immune dysfunction and hematologic diseases. Increased Proteobacteria and related taxa have been reported in MDS patients, especially those with high-risk disease [17,18], where dysbiosis has been linked to aberrant immune activation. Similarly, in IEI with gastrointestinal involvement—such as CTLA-4 deficiency [14], chronic granulomatous disease [15], Wiskott–Aldrich syndrome [23], or common variable immunodeficiency [16,24]—an expansion of Proteobacteria and other inflammatory taxa alongside reduced beneficial commensals has been described [13].

Interestingly, post-HSCT GATA2-deficient patients did not exhibit the above GM alterations, showing instead GM profiles much closer to HD. This observation suggests that immune reconstitution may contribute to a stabilization of GM composition, although causality cannot be established. The dysbiosis observed in non-HSCT patients may be associated with the underlying hematopoietic and immune dysfunction present prior to transplantation, rather than representing a fixed, germline-driven feature of GATA2 deficiency. However, transplantation-related factors may also contribute to the observed patterns and cannot be excluded [25].

To investigate the functional consequences of the microbiome differences, we analyzed fecal metabolomes and lipidomes in a subset of GATA2-deficient patients. The results suggested metabolic shifts associated with the dysbiotic microbiome in non-transplanted patients, and a partial normalization after HSCT that parallels the microbiota findings. Untargeted metabolic profiling of fecal samples showed that GATA2-deficient patients have a distinct fecal metabolic fingerprint compared to healthy individuals. Non-HSCT GATA2 patients had significantly elevated levels of several metabolites linked to inflammation and altered GM activity. For instance, we observed increases in secondary bile acids (e.g., deoxycholic acid, lithocholic acid) and long-chain fatty acids (e.g., palmitoleic acid, adrenic acid), which can be microbial or host-derived and are known to influence gut and immune homeostasis, ultimately promoting inflammation [26,27,28]. There were also higher levels of acylcarnitines (e.g., decenoylcarnitine) and carnitine, suggesting an upregulation of fatty acid oxidation pathways and mitochondrial metabolism in the gut environment associated with the expansion of Enterobacteriaceae [29]. Additionally, pro-inflammatory or bioactive lipids like sphingosine were elevated. These changes may suggest a metabolic profile enriched in features previously associated with inflammatory states in the gut of non-HSCT patients [30]. Conversely, several potentially beneficial metabolites were reduced in non-HSCT GATA2 patients. Notably, we found lower concentrations of short-chain fatty acids, such as valeric acid, along with decreased levels of specific amino acid metabolites (e.g., S-propyl-cysteine, leucyl-glutamate), compared to HD. Short-chain fatty acids are key microbial fermentation products that normally help regulate inflammation, gut barrier integrity, and immune cell function [31,32].

Fecal lipidomics further supported these observations. Although the overall lipidome profile did not significantly differ between HD and GATA2 patients, several pro-inflammatory or energy-related lipid classes were enriched in non-HSCT patients, with partial recovery after HSCT. Triglycerides, phosphatidylcholines, sphingomyelins, hexosylceramides (complex sphingolipids often involved in cell signaling and inflammation), and acylcarnitines were increased in the non-HSCT group, echoing the metabolomic evidence of enhanced fatty acid oxidation [33,34]. In contrast, anti-inflammatory or structural lipids such as certain phospholipid subclasses were reduced, such as significantly lower levels of lysophosphatidylethanolamine, lysophosphatidylglycerol, and lysophosphatidylinositol in non-HSCT patients relative to both HD and post-HSCT patients [35,36]. The net effect of these lipidomic changes may result in a gut milieu skewed towards inflammation, enhanced lipid oxidation, and membrane remodeling in active GATA2 deficiency, although their biological significance requires further investigation. As the fecal lipidome for the above classes was closer to HD, it appears that HSCT may be associated with partial normalization of these features.

Taken together, the metabolomic and lipidomic alterations appear to parallel microbiome findings. Non-HSCT patients exhibit a GM enriched in LPS-associated bacteria, alongside a metabolite profile including inflammatory lipids, bile acids, and oxidative metabolites that have been previously associated with inflammatory states. Post-HSCT patients showed a trend toward more homogeneous and partially normalized profiles. These observations raise the possibility of an interaction between microbiome composition and metabolic output, although the directionality and causality of this relationship cannot be determined in this study. Importantly, the more homogeneous metabolic and lipidomic profiles observed in post-HSCT patients may reflect the combined effects of immune reconstitution and transplantation-related factors. The relative contribution of these factors cannot be disentangled in the current study.

The link between microbiome, metabolism, and mitochondria is an intriguing avenue for future research. Mitochondrial oxidative stress has been implicated in the premature evolution to MDS/AML in GATA2 deficiency [37]. Alterations in the GM and a pro-oxidative fecal metabolome may represent an additional layer of association between microbial-derived metabolites and cellular metabolic pathways.

Our study has several limitations. First, the sample size was small, which limits the statistical power and generalizability of the findings. GATA2 deficiency is a rare disease, and recruiting large cohorts is challenging, but future multicenter studies will be needed to confirm our observations across a broader population. Second, the study design is cross-sectional, which precludes any inference on temporal relationships or causality. Therefore, we cannot definitively establish whether the dysbiosis we observed actively contributes to clinical deterioration or if it is purely a byproduct of the disease process. Longitudinal studies—sampling the microbiome and metabolome before and after interventions like HSCT or during disease progression—would help clarify temporal relationships and causation. Third, uncontrolled confounding factors could have influenced the GM in our cohort. The microbiome is highly sensitive to diet, medications, geography, lifestyle, and other environmental factors. While our patients and controls were age-matched, had no active infections, and were not taking medications at the time of the study, we did not control for diet or smoking, for instance. It is possible that some differences or lack thereof in microbial diversity were influenced by these external factors rather than by GATA2 status alone and should be considered when interpreting our results. Additionally, factors like geographical location and ethnicity can shape microbiome composition. Although our study cohort was relatively homogeneous in this respect, minimizing heterogeneity, replication in other populations is still needed. An additional limitation is the lack of fully matched multi-omics data at the individual level, as metabolomic and lipidomic analyses in HD were performed on samples different from those used for microbiome profiling. This precluded integrative analyses (e.g., correlation matrices) across datasets. In addition, clinical heterogeneity represents an important source of variability. GATA2 deficiency encompasses a broad spectrum of disease manifestations and stages, which may differentially impact microbiome composition and metabolic output. The limited sample size prevented stratified analyses and may mask disease stage–specific effects. Furthermore, transplantation-related factors may influence both microbiome and metabolomic profiles. Although post-HSCT patients included in this study had no chronic GVHD and were off immunosuppressive therapy, prior conditioning regimens and long-term immune reconstitution may still contribute to the observed differences. The relative contribution of HSCT-related factors versus disease resolution cannot be disentangled in this study. GATA2 deficiency has systemic effects, and it would be insightful to determine if the metabolomic disturbances in stool are mirrored in the bloodstream or the bone marrow microenvironment, which were not analyzed in this study. Thus, it remains to be confirmed whether the elevated inflammatory metabolites (e.g., bile acids or fatty acids) also circulate systemically at higher levels in non-HSCT patients, potentially acting directly on bone marrow cells. Taken together, these limitations highlight the challenges of interpreting microbiome data in rare and heterogeneous genetic conditions and reinforce the descriptive nature of the present study.

Despite these limitations, our study represents an initial descriptive and exploratory investigation of the microbiome–metabolome–lipidome axis in GATA2 deficiency. Our findings suggest the presence of compositional and metabolic patterns associated with disease or treatment status, which may inform the design of future studies. Although preliminary, these observations support further investigation in larger, longitudinal and better-controlled cohorts to clarify the role of the microbiome in GATA2 deficiency and its potential clinical relevance.

## 4. Materials and Methods

### 4.1. Study Design and Sample Collection

Patients, parents or the legal representative provided informed consent to be part of the study, approved by the Ethics Committee of Comitato Etico Territoriale Lombardia 3 (GATA2.IT.REG, 15 October 2025), and conducted in accordance with the International Conference on Harmonization (ICH) for Good Clinical Practice (GCP) and with the Declaration of Helsinki. All patients included in the study had a confirmed diagnosis of GATA2 deficiency based on the identification of pathogenic or likely pathogenic germline variants in the *GATA2* gene. Patients were consecutively recruited at participating centers during routine clinical follow-up. Disease stage at the time of sampling was recorded and categorized as asymptomatic, cytopenia, bone marrow failure, MDS, or prior AML in remission in non-HSCT patients, while transplanted individuals were classified as post-HSCT. Classification was based on established diagnostic criteria [38]. Inclusion criteria required the absence of antibiotic treatment in the preceding three months. Additional criteria for the post-HSCT group included the absence of chronic graft-versus-host disease (GVHD), full donor chimerism, and no immunosuppressive treatment for at least six months prior to enrollment. Twenty-four age- and sex-matched healthy individuals (HDs) were recruited as controls. While detailed dietary or behavioral (smoking or lifestyle) data were not collected, participants were enrolled from the same geographic region (Italy) and recruited contemporaneously to patient sampling to minimize environmental variability.

### 4.2. 16S rRNA Gene Sequencing

Microbial DNA was extracted from 200 mg of fecal sample using QIAmp Fast DNA Stool Mini Kit (Qiagen, Hilden, Germany). Next-generation sequencing (NGS) of the bacteria-specific 16S ribosome gene was performed utilizing a microbiota solution B kit—hypervariable regions V3-V4-V6 (Arrow Diagnostics S.r.l., Genoa, Italy). An Illumina MiSeq Platform (Illumina Inc., San Diego, CA, USA) was used for sequencing. Taxonomic assignment and a bioinformatic analysis were performed using the MicrobAT (Microbiota Analysis Tool; SmartSeq S.r.l., Novara, Italy). No missing data were present. Only samples passing quality control were included.

### 4.3. Statistical Analysis

Data analyses were performed using R software version 4.4.2 (R Foundation for Statistical Computing, Vienna, Austria). Categorical variables are presented as frequencies (percentages), and continuous variables are presented as means and standard errors. Microbiome analyses were conducted on species-level relative abundance data. Alpha diversity was assessed using Shannon and Simpson indices and Pielou’s evenness, and group differences were evaluated using the Wilcoxon rank-sum test. Beta diversity was calculated using Bray–Curtis and Jaccard distances and visualized using principal coordinates analysis (PCoA); group differences were tested using the Permanova test. Differential abundance across groups was assessed using Kruskal–Wallis tests followed by pairwise Wilcoxon tests, with false discovery rate (FDR) correction. *p*-values less than 0.05 in a two-tailed test were considered statistically significant.

### 4.4. Metabolomic and Lipidomic Analyses

#### 4.4.1. Sample Extraction

Polar metabolites and lipids were extracted from all fecal samples as previously described [39] in non-HSCT (n = 3) and post-HSCT (n = 2) GATA2-deficient patients and compared with healthy donors (HD; n = 5). Briefly, 125 ul of water (H_2_O) was added to 250 mg of the sample, which was then vortexed and sonicated for 15 min. Afterwards, 250 ul of acetonitrile (ACN) was added, and the samples were vortexed and centrifuged at 15,000 RPM for 3 min at 4 °C. The supernatant was separated from the pellet, split into equal volumes to analyze separately polar metabolites and lipids, dried, and finally reconstituted as follows: 200 μL of ACN:H_2_O (50:50 *v*/*v*) for polar metabolites analysis, and 200 μL of pure isopropanol (ISO) for lipids analysis. Quality control samples (QC samples) were prepared by pooling 10 μL of each sample in a single Eppendorf tube.

#### 4.4.2. UHPLC-MS Analysis

Samples were analyzed as previously described [40] using a UHPLC-MS platform, which includes an Agilent 1290 II liquid chromatography system coupled to a quadrupole-time-of-flight mass spectrometer (Q-TOF—Agilent Technologies, Palo Alto, CA, USA). Each sample was analyzed in three technical replicates. The chromatographic separation for polar analytes was achieved using an InfinityLab Poroshell 120 HILIC-Z (2.1 × 150 mm 2.7 μm) column; the chromatographic eluents were: A, 100% 20 mM ammonium acetate and 5 μM medronic acid; and B, 100% ACN. The gradient applied was: 0 min 90%B, 1 min 90%B, 8 min 78%B, 12 min 60%B, 15 min 10%B, 18 min 10%B, and 23 min 90%B at a flow rate of 0.4 mL/min. The mass spectrometer operates at a resolution of 40,000 FWHM with a full scan range of 40−1200 *m*/*z* in both positive and negative polarity. On the other hand, the lipidome separation was achieved with an ACQUITY UPLC CSH C18 column (2.1 × 100 mm, 1.7 μm-Waters, Milford, MA, USA). The mobile phases for chromatographic separation were: A, 10 mM ammonium acetate:ACN 40:60 *v*:*v* with 0.1% acetic acid; and B, ISO:phase A 90:10 *v*:*v*. The elution gradient was: 0 min 99% A, 1 min 99% A, 1.10 min 60% A, 5 min 20% A, 11 min 20% A, 12 min 1% A, 18 min 1% A, 18.10 min 60% A, and 20 min 99% A at a flow rate of 0.25 mL/min. The resolution of the mass spectrometer was set at 50,000 FWHM, and it operated in the full scan range of *m*/*z* 100−1350. The samples were analyzed in full scan mode in triplicate, both in positive and negative ionization modes. QCs were used to monitor the performance of the analysis and were injected every 5 samples. At the end of the analysis, 6 injections of QCs were used to collect MS/MS spectra in data-dependent mode (DDA) using an iterative approach. In this method, if an ion is fragmented in the first run, it will be ignored in the subsequent runs if RT and MS parameters are respected; otherwise, it will be re-fragmented. This approach overcomes the principal DDA defect, which usually fragments only the most intense ions, and permits the fragmentation of low-intensity ions and a deeper analysis. No missing data were present. Only samples passing quality control were included.

#### 4.4.3. Data Analysis

MassHunter Profinder (Agilent Technologies, Palo Alto, CA, USA) was used for batch-processing feature integration and annotation using LC/MS Personal Compound Databases (PCDL). The library for polar metabolites and lipids was built based on accurate mass, MS/MS, isotopic pattern and retention time, and using online databases such as HMDB [10.1093/nar/gkab1062] and METLIN [10.1021/acs.analchem.7b04424]. The samples analyzed in full scan were matched based on mass formula extraction and retention time against the built library.

Metaboanalyst 6.0 was used for univariate and multivariate statistical analyses [41]. Data were normalized by the sum of the features and then Log10 transformed before statistical analyses. Volcano Plots were obtained using a *p*-value threshold of 0.05 and a fold change threshold of ±1.5. Functional analysis on polar metabolites was performed by using the Small Molecule Pathway Database (SMDB) [10.1093/nar/gkp1002].

## 5. Conclusions

This exploratory study describes differences in GM composition, as well as metabolomic and lipidomic profiles, in GATA2-deficient patients. Non-HSCT individuals showed a relative enrichment of taxa, metabolites, and lipids previously associated with inflammatory processes, suggesting the presence of an altered gut environment accompanied by metabolic changes. These alterations, particularly involving lipid metabolism, energy pathways, and oxidative processes, may reflect features associated with the immune and hematopoietic dysfunction observed in GATA2 deficiency. However, given the limited sample size and cross-sectional design, these findings should be interpreted with caution and require validation in larger, well-characterized cohorts.

## Figures and Tables

**Table 1:** Demographic and immune-hematological characteristics of enrolled patients.

	P1	P2	P3	P4	P5	P6	P7	P8	P9	P10	P11	P12
Age	28	20	21	28	18	24	27	20	20	23	25	47
Gender	F	M	M	M	M	F	F	M	F	F	F	M
Variant	c.1009C>T; p.Arg337X	c.257_258delGC; p.Cys85fs	c.112C>T; p.Gln38X	c.1084C>T; p.Arg362X	c.G1079A; p.Trp360X	c.1084C>T; p.Arg362X	Intron 4 deletion	c.919C>T; p.Arg307Trp	c.503_504insGCTC; p.His169Leufs*17	c.1017+572C>T	c.380_383dupACC; p.Ser129Profs*57	c.503_504insGCTC; p.His169Leufs*17
HSCT	Yes	Yes	Yes	Yes	Yes	Yes	No	No	No	Yes	No	No
Time from HSCT, years	16	12	3.5	9	5	8	-	-	-	6	-	-
Disease stage at sampling	Post-HSCT	Post-HSCT	Post-HSCT	Post-HSCT	Post-HSCT	Post-HSCT	Prior AML (at 13 yo) in remission	Asymptomatic	MDS-LB	Post-HSCT	MDS-LB	Asymptomatic
Reason for HSCT	MDS-LB	MDS-LB	BMF	Immunodeficiency	BMF	MDS-EB	-	-	-	MDS-EB	-	-
Hb	13.6	15	11.2	15.1	13.5	12.1	14.1	17.5	13.3	11.8	12	17.2
WBC, mmc	6430	4550	3220	4950	4330	5110	6480	6150	3220	7470	3350	8290
ANC, mmc	3280	2350	1810	2410	2450	3090	2310	2900	1480	4360	1530	5490
ALC, mmc	2420	1780	830	1920	1360	1640	3720	2400	1620	2580	1650	1850
AMC, mmc	490	220	210	440	NA	240	NA	560	30	920	30	830
PLT, mmc	254,000	179,000	159,000	215,000	181,000	284,000	130,000	201,000	204,000	233,000	180,000	255,000
CD3+, mmc	1470	1320	NA	NA	NA	NA	NA	1140	1460	2070	1470	1720
CD4+, mmc	800	670	270	680	570	NA	NA	640	720	1070	590	1200
CD8+, mmc	590	530	220	660	400	NA	NA	430	650	860	740	490
CD19+, mmc	220	310	170	260	140	NA	NA	420	60	340	60	180
CD16+CD56+, mmc	250	90	110	150	160	NA	NA	50	200	150	70	140
IgG, mg/dL	1210	870	1370	1163	659	913	673	746	884	571	1282	1090
IgA, mg/dL	104	170	4	304	16	4	50	136	51	111	112	155
IgM, mg/dL	51	56	142	215	35	144	50	98	92	42	171	58

ALC = absolute neutrophil count. AMC = absolute monocyte count. AML = acute myeloid leukemia. ANC = absolute neutrophil count. F = female. Hb = hemoglobin. HSCT = hematopoietic stem cell transplantation. M = male. MDS-EB = Myelodysplastic syndrome with excess blasts. MDS-LB = Myelodysplastic Syndrome with Low Blasts. PLT = platelet count. WBC = white blood cells.

## Data Availability

The data are available upon reasonable request from the authors.

## Supplementary Materials

The following supporting information can be downloaded at: https://www.mdpi.com/article/10.3390/ijms27104294/s1.

## Abbreviations

The following abbreviations are used in this manuscript:

GM	Gut microbiota
GVHD	Graft-versus-host disease
HD	Healthy donors
HSCT	Hematopoietic stem-cell transplantation
IEI	Inborn errors of immunity
MDS	Myelodysplastic syndrome
