# Supplementary material for: A Gut Feeling: An Exploratory Multi-Omics Study of Gut Microbiome Dysbiosis and Metabolome and Lipidome Alterations in GATA2 Deficiency
Source: Int J Mol Sci. 2026 May 12;27(10):4294. doi: 10.3390/ijms27104294 (PMC13207708; doi:10.3390/ijms27104294)

A Gut Feeling: An Exploratory Multi-Omics Study of Gut Microbiome Dysbiosis and Metabolome and Lipidome Alterations in GATA2 Deficiency

Samuele Roncareggi [1], Francesca Fioredda [2], Katia Girardi [3], Simone Serrao [1], Giulia Capitoli [4,5], Rebecca Fumagalli [1], Marta Nobile [1], Grazia Fazio [1,6], Fabiola Guerra [7], Maria Grazia Valsecchi [4,5], Stefano Rebellato [6], Marika Casillo [8], Maria Rosaria Fantuz [8], Giovanni Savarese [8], Giuseppe Paglia [1], Eleonora Gambineri [9,10], Adriana Cristina Balduzzi [1,7], Andrea Biondi [6] and Francesco Saettini [6,7,*] on behalf of the Italian GATA2 Study Group

[1] Dipartimento di Medicina e Chirurgia, Università degli Studi Milano-Bicocca, 20900 Monza, Italy

[2] Unit of Hematology, Istituto di Ricovero e Cura a Carattere Scientifico (IRCCS) G. Gaslini, 16147 Genoa, Italy

[3] Department of Pediatric Hematology/Oncology, Cell and Gene Therapy, Istituto di Ricovero e Cura a Carattere Scientifico (IRCCS) Bambino Gesù Children's Hospital, 00165 Rome, Italy

[4] Bicocca Bioinformatics Biostatistics and Bioimaging (B4) Centre, School of Medicine and Surgery, University of Milano-Bicocca, 20126 Monza, Italy

[5] Biostatistics and Clinical Epidemiology, Fondazione Istituto di Ricovero e Cura a Carattere Scientifico (IRCCS) San Gerardo dei Tintori, 20900 Monza, Italy

[6] Centro Tettamanti, Fondazione Istituto di Ricovero e Cura a Carattere Scientifico (IRCCS) San Gerardo dei Tintori, 20900 Monza, Italy

[7] Pediatria, Fondazione Istituto di Ricovero e Cura a Carattere Scientifico (IRCCS) San Gerardo dei Tintori, 20900 Monza, Italy

[8] Ames Centro Polidiagnostico Strumentale, 80013 Casalnuovo di Napoli, Italy

[9] Department of Pediatric Oncology/Hematology, Meyer Children's Hospital Istituto di Ricovero e Cura a Carattere Scientifico (IRCCS), 50139 Florence, Italy

[10] Department of Neurosciences, Psychology, Drug Research and Child Health (NEUROFARBA), University of Florence, 50139 Florence, Italy

* Correspondence: f.saettini@gmail.com

Supporting information

Table S1. Demographic and immune-hematological characteristics of enrolled patients.

	Non-HSCT group	HSCT group	P value
Number of patients	5	7	
Gender, male	2	3	>0.99
Age, median (range)	24 years (19-46)	22 years (19-28)	0.39
Complete blood count with differential, median (range)			
Hemoglobin g/dl	14.1 (12.0-17.5)	13.5 (11.2-15.1)	0.24
White blood cells/mmc	6150 (2840-6520)	4550 (3220-7470)	0.97
Lymphocytes/mmc	2000 (1250-3720)	1778 (830-2582)	0.38
Neutrophils/mmc	2310 (1480-3590)	2450 (1810-4360)	0.39
Monocytes/mmc	30 (0-800)	340 (210-920)	0.5
Eosinophils/mmc			
Platelets/mmc	201000 (130000-274000)	233000 (159000-284000)	0.3
Lymphocyte subsets/mmc, median (range)			
CD3+	1464 (1137-4069)	1394 (1234-2079)	0.56
CD4+	720 (492-1196)	675 (265-1067)	0.37
CD8+	615 (430-2525)	559 (216-858)	0.31
CD19+	58 (0-419)	241 (133-343)	0.24
CD16+CD56+	59 (0-202)	149 (92-250)	0.17
Immunoglobulin mg/dl, median (range)			
IgG	815 (673-1282)	913 (571-1366)	0.71
IgA	82 (50-136)	104 (4-304)	0.81
IgM	95 (50-171)	56 (35-215)	0.91

Figure S1. Volcano Plot of Differentially Abundant Metabolites in Non-HSCT GATA2-Deficient Patients Compared with Healthy Individuals

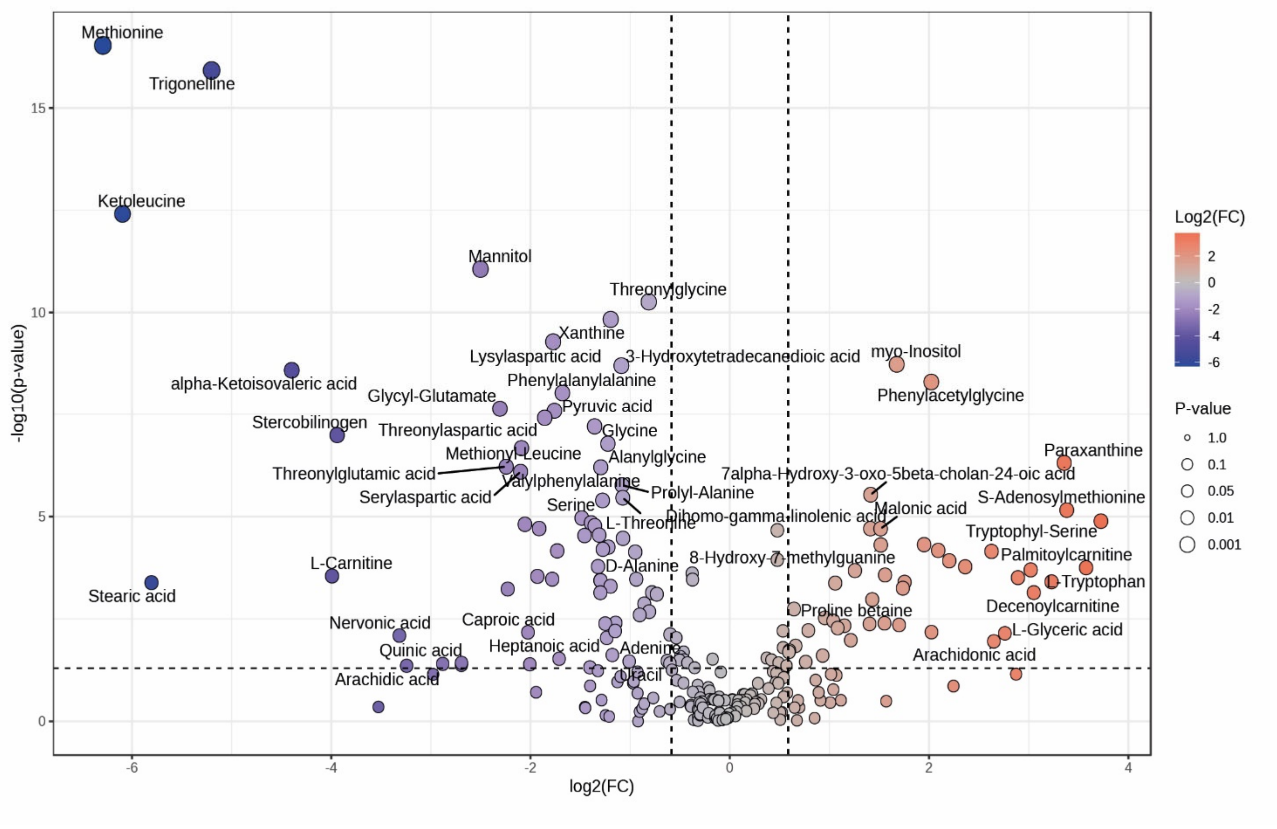

Supplement: Supplementary file 1 [file ijms-27-04294-s001.zip › ijms-4206790-supplementary.pdf]
